# Development and application of a stepwise-assembled modular biomimetic lung chip for analyzing formaldehyde-induced cellular ferroptosis

**DOI:** 10.3389/fbioe.2025.1570270

**Published:** 2025-06-25

**Authors:** Siyu Chen, Zezhi Li, Quanping Yan, Chenfeng Hua, Pingping Shang, Kejian Liu, Junwei Zhao, Guangxiang Jin, Xiang Li, Fuwei Xie

**Affiliations:** ^1^ Key Laboratory of Tobacco Chemistry, Zhengzhou Tobacco Research Institute of CNTC, Zhengzhou, China; ^2^ Beijing Life Science Academy, Beijing, China; ^3^ Technology Center, China Tobacco Guangdong Industrial Co., Ltd., Guangzhou, China

**Keywords:** microfluidic, modular, stepwise-assembled, biomimetic lung chip, formaldehyde, ferroptosis

## Abstract

Formaldehyde poses a significant threat to human health, yet its toxicity assessment is limited by current detection methods. This study developed a modular biomimetic lung chip which consists of gas concentration gradient generator module and cell culture module, assembled using alignment holes and micropillars. This design enables stepwise experimental procedures, including cell loading, gas concentration gradient exposure, and cell sample collection, through the assembly and disassembly of the modules. Using this chip to investigate the formaldehyde-induced cellular ferroptosis, BEAS-2B cells were exposed to formaldehyde under a concentration gradient. Transcriptomic sequencing revealed the effects of different formaldehyde concentrations on the expression of ferroptosis-related genes in cells, identifying 12 ferroptosis-related genes (FRGS) and an enhancement of oxidative stress. The ferroptosis inhibitor (deferoxamine mesylate) significantly reduced cell death and reactive oxygen species levels, confirming the involvement of ferroptosis in formaldehyde-induced toxicity. Furthermore, deferoxamine mesylate modulated the expression of hub genes of FRGS, including *PTGS2*, *ATF3*, *CDKN1A*, *PLIN2*, and *DUOX1*, at both transcript and protein levels. These findings provide novel insights into the molecular mechanisms underlying formaldehyde-induced respiratory damage and establish the modular biomimetic lung chip as a powerful platform for studying environmental toxicants.

## Introduction

Over the past few decades, advancements in microfabrication technology have driven the miniaturization and functional integration of microfluidic chips (Pan et al., 2022; Zhao et al., 2023). Constructing a functional microfluidic chip requires addressing several challenges, including the biocompatibility and optical properties of materials, fabrication precision, integration of necessary experimental functions, and cost (Quintard et al., 2024; Salvador-Morales and Grodzinski, 2022; Thompson et al., 2020). Modular chip design presents a promising solution to these issues (Carvalho et al., 2024; Lai et al., 2022).

The core design concept behind modular chips is to decompose complex chip systems into independent functional modules (Fernandes et al., 2018; Lai et al., 2022). Each module can be individually designed, manufactured, tested, and then integrated as needed. Modular design has already been applied in microfluidic chips. For instance, Rhee’s group pioneered the conception and development of a modular assembly platform, employing the innovative use of prefabricated polydimethylsiloxane (PDMS) chips (Rhee and Burns, 2008). Through Lego-like building blocks, they created a platform capable of liquid diversion and convergence. Similarly, Langelier and colleagues posited a concept for a multifunctional chip module, one which integrates valves, pumps, mixers, and other active components, creating a modular platform designed like a puzzle to achieve precise control over liquid flow (Langelier et al., 2011). This modularity enables researchers to carry out sequential experimental steps—such as mixing, extraction, separation, reaction, and purification—on different liquid samples. However, despite its success in microfluidics, modular design has not yet been widely applied in organ chips (Carvalho et al., 2024).

As the demand for toxicity assessments increases, traditional cell biology experiments can no longer meet the high fidelity required for certain tasks. Organ chips based on microfluidic technology provide a novel solution to meet the demands of *in vitro* experiments. Zhang et al. developed a highly biomimetic lung chip that successfully simulated the alveolar-capillary dual tissue interface and its physiological microenvironment by constructing a three-dimensional co-culture alveolar chip system based on microfluidic technology (Zhang et al., 2020). Their study revealed how alveolar epithelial cells and microvascular endothelial cells enhance chemical defense through synergistic interactions when stimulated by pollutants such as benzo [a]pyrene and nicotine. Similarly, Zheng et al. developed a high-throughput biomimetic lung chip equipped with a concentration gradient generation unit (Zheng et al., 2017). Combining transcriptomics with microfluidic chip technology, their research demonstrated that PM2.5 induces carcinogenic traits, such as apoptosis evasion, angiogenesis, and abnormal proliferation, in BEAS-2B cells through the activation of signaling pathways like PI3K-Akt, FGF/FGFR/MAPK/VEGF, and JAK-STAT. Despite these advances, achieving both high biomimetic performance and high-throughput testing has often been challenging due to limitations in the integration capacity of chip functional units. Recently, the application of modular microfluidic chips in organ biomimicry has gained prominence, offering new insights into resolving the compatibility between biomimetic performance and testing throughput (Carvalho et al., 2024; Wu et al., 2023). By decomposing functional units into independent modules, modular design enables stepwise assembly tailored to experimental needs. This approach reduces the complexity of chip design and fabrication, enhancing both flexibility and operability (Koh and Hagiwara, 2024; Parandakh et al., 2023). It also makes microfluidic chips more accessible and customizable for various experimental applications.

This study presents the development and application of a modular biomimetic lung chip for investigating cellular ferroptosis induced by formaldehyde exposure. The chip separated the gas concentration gradient generator unit from the cell culture unit, enabling the sequential execution of key experimental procedures—such as cell seeding, gas exposure, and collection of cellular samples—through the assembly and disassembly of individual modules. Furthermore, the modular lung chip was utilized to conduct formaldehyde gas exposure experiments in conjunction with transcriptomic analysis to examine alterations in ferroptosis-related molecules under varying formaldehyde concentrations. *In situ* fluorescence detection and reverse transcription-quantitative PCR (RT-qPCR) were employed to validate the expression patterns of ferroptosis biomarkers, correlating with transcriptomic data across different exposure levels.

## Results

### The modular biomimetic lung chip and its application process

**FIGURE 1:**
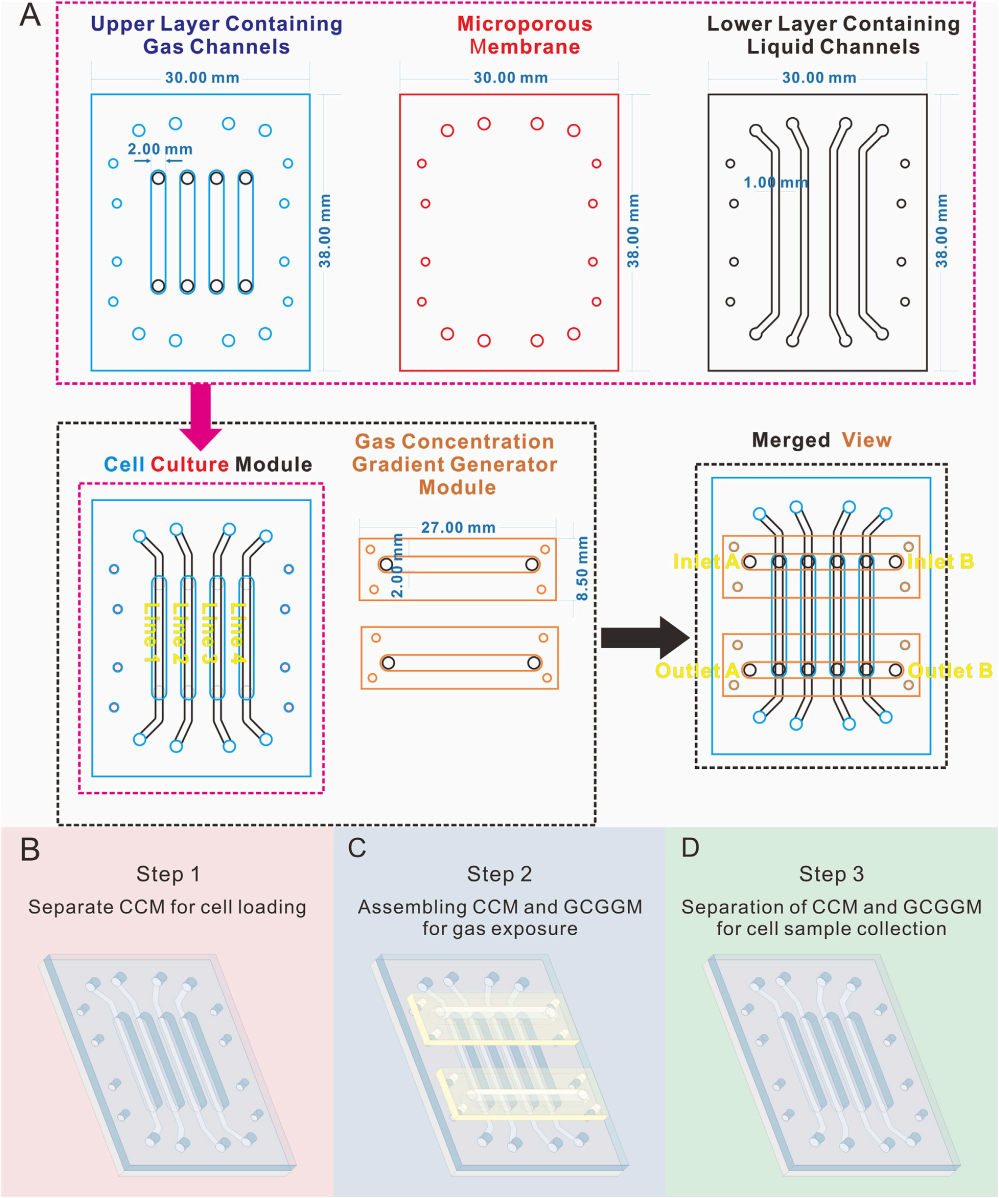
Diagram illustrating the geometric parameters and operational workflow of the modular biomimetic lung chip. **(A)** Schematic and geometric parameters of the layers and modules; **(B–D)** Illustration of the stepwise assembly process of the chip modules in the form of a schematic diagram.

**FIGURE 2:**
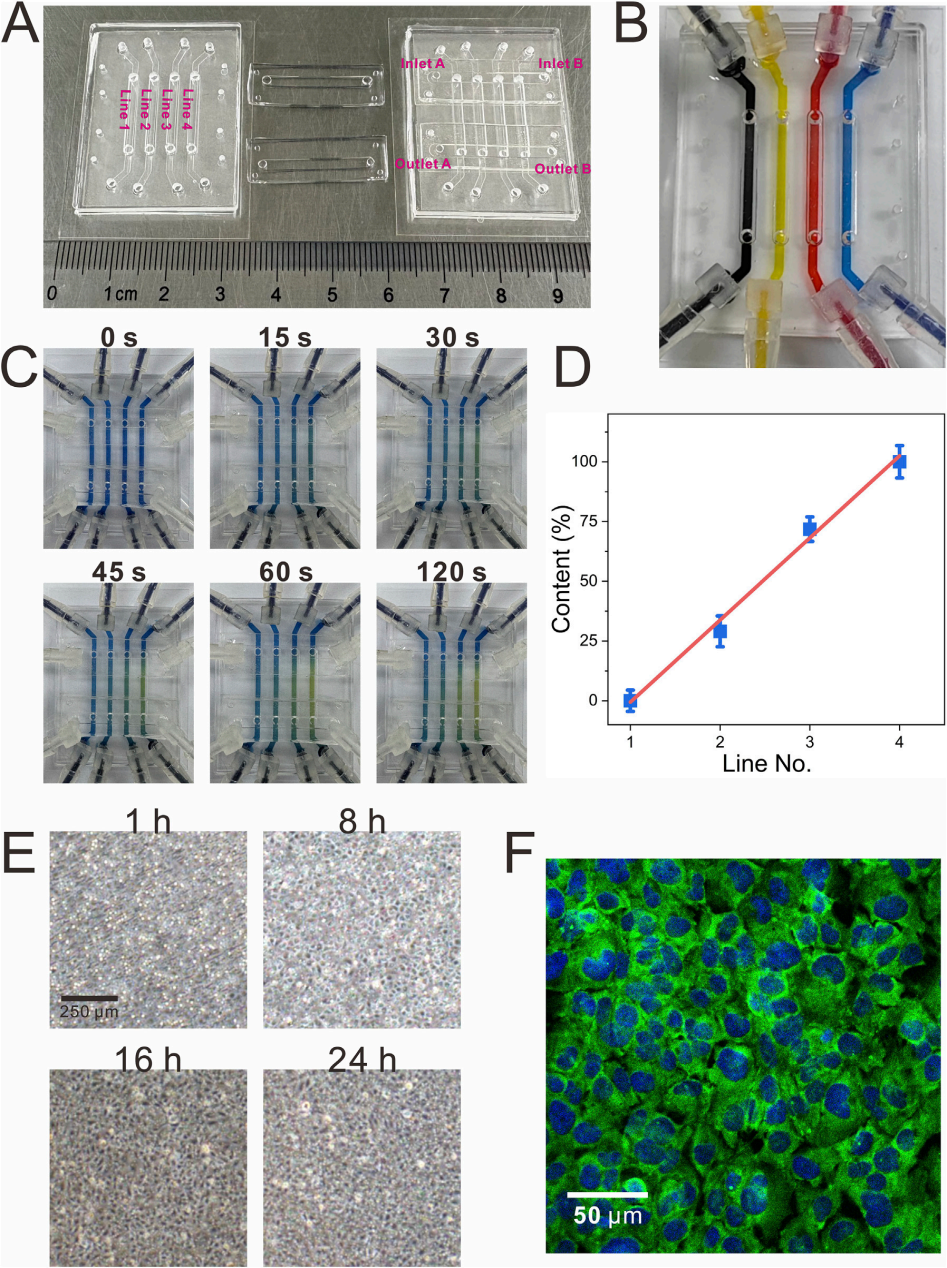
Visualization of the functional validation of the modular biomimetic lung chip. **(A)** The physical representation of the fully assembled modular chip; **(B)** The image verifying the independence of liquid channels during the establishment of the gas-liquid interface using inks of different colors; **(C,D)** Visualization of the gas concentration gradient through the colorimetric reaction of CO_2_ and bromothymol blue, along with a linear analysis of the color intensity; **(E)** Dynamic monitoring of cell growth within the chip over a 24-h period; **(F)** Graphical representation of immunocytochemical characterization of tight junctions (ZO-1, green; DAPI, blue) in BEAS-2B cells.

As illustrated in Figures 1, 2A, the cell culture module (CCM) of the biomimetic lung chip incorporates four parallel gas channels in the center of the upper layer, enabling stepwise cell loading, gas exposure, and sample collection through modular assembly and disassembly. The lower layer contains four parallel liquid channels designed to facilitate the continuous perfusion of culture medium, ensuring sufficient cell nutrition during experiments. Four alignment apertures are located on each horizontally oriented lateral side of these channels. The liquid channels and alignment apertures are precisely aligned with those in the upper layer, enabling seamless integration. A microporous membrane is situated between the two layers of the CCM, providing support for cell attachment and establishing a gas-liquid interface. The gas concentration gradient generator module (GCGGM) comprises a single horizontal channel flanked by alignment micro-pillars, enabling rapid self-alignment with the corresponding apertures on the upper layer. During gas exposure experiments, two GCGGMs are attached to the inlet and outlet of the upper layer’s gas channels using pressure-sensitive adhesive. Two different gases are introduced into the GCGGM inlets (inlet A and B), diffuse within the horizontal channel, and are drawn into the upper layer’s gas channels by vacuum pressure, establishing a consistent gas concentration gradient (Figure 1A).

The stepwise assembly of chip modules addresses various experimental needs. During the cell loading phase, only the CCM is required (Figure 1B). Cells are injected into the gas channels of the CCM, which accelerates cell loading compared to previous designs that integrated the gas concentration gradient generation unit (Li et al., 2024). This configuration is intended to ensure uniform distribution of cells at the polycarbonate (PC) microporous membrane. In the course of the gas exposure phase, the CCM is assembled with the GCGGM, with the gas channels of the GCGGM being connected to those of the CCM for the purpose of establishing a complete gas concentration gradient generator unit (Figure 1C). After the gas exposure experiment, the CCM and GCGGM can be separated, enabling the collection of cell samples from the different gas channels of the CCM during the sample collection phase (Figure 1D).

### Visual characterization of chip functions

To evaluate the functionality of the chip, different colored inks were introduced into each liquid channel of the CCM (Figure 2B). This approach ensured that liquid did not overflow through the microporous membrane into the gas channels in the course of the establishment of the gas-liquid interface, thereby verifying the independence of all liquid channels. Furthermore, the gas concentration gradient function was visualized using CO_2_ and bromothymol blue, a pH indicator. When dissolved in the indicator, CO_2_ forms carbonic acid, causing a color shift from blue to yellow; higher CO_2_ concentrations produce a more intense yellow hue. As illustrated in Figures 2C,D, air and CO_2_ were introduced into the two inlets of the GCGGM. The gradual accumulation of CO_2_ led to a progressive color change in the pH indicator, which eventually stabilized, confirming the successful formation of the gas gradient.

### Visual characterization of chip functions

After loading, it was imperative to statically culture the cells to ensure robust adhesion to the PC microporous membrane. During this period, cell growth was closely monitored, and confluence was achieved between 16 and 24 h (Figure 2E). Once confluence was reached, aspirating the medium from the gas channels of the upper chip and introducing fresh medium into the liquid channels of the lower chip was performed using a syringe pump. Following an additional 24 h of continuous dynamic culture at the air-liquid interface, immunofluorescence detection of the tight junction protein ZO-1 demonstrated its pronounced accumulation at intercellular contacts, indicating robust cell-cell adhesion (Figure 2F).

### Validation of the formaldehyde exposure system

**FIGURE 3:**
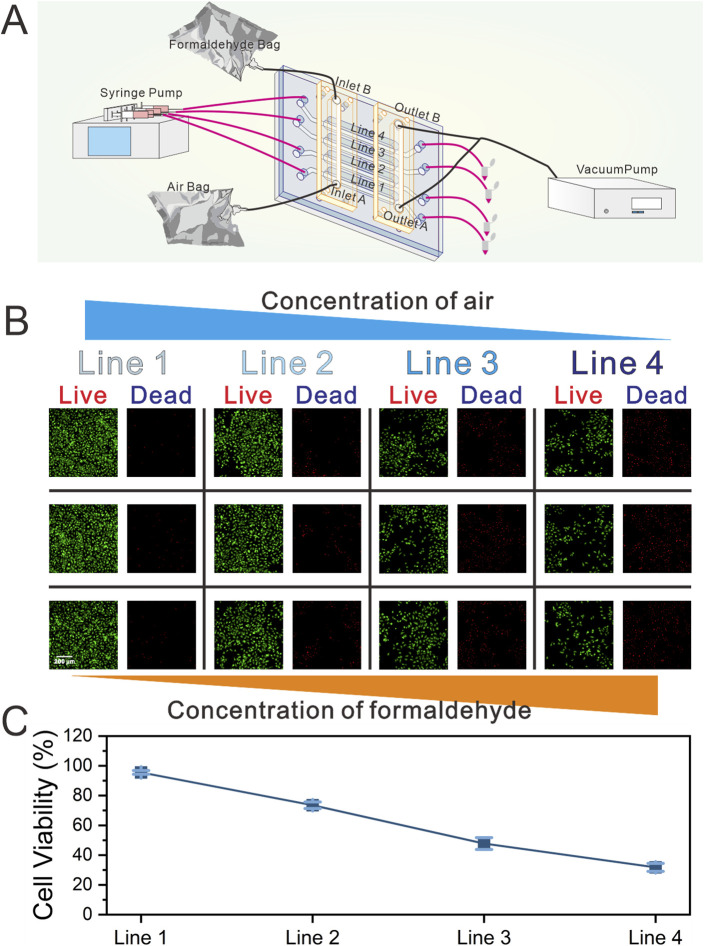
Schematic of the real-time gas exposure system and gas exposure functional validation experiment. **(A)** Schematic diagram of the composition and operational functions of the gas exposure system (the gas exposure system includes the biomimetic lung chip, high-precision vacuum pump, four-channel syringe pump, and two gas sampling bags) **(B)** Representative images demonstrating on-chip cell viability following formaldehyde exposure, as determined by the Live/Dead assay (green representing live cells, and red representing dead cells). And the three images in the same row represent images from random positions within the corresponding Line.; **(C)** Quantification of cell viability.

Once the cells on the chip reached confluence, the culture medium within the gas channels were carefully aspirated using a pipette. After 2 h of continuous dynamic cultivation, formaldehyde gas exposure was initiated (Figure 3A). The formaldehyde gas exposure setup included a high-precision vacuum pump, a four-channel syringe pump, and two 4 L gas bags. One gas bag was filled with 0.4 mL of formaldehyde solution and 2 L of sterile air, equilibrated for 2 h at 25 °C. The second bag contained 2 L of sterile air. The air and formaldehyde bags were linked to inlets A and B of the GCGGM, respectively. Fresh medium was continuously infused into the liquid channels at a flow rate of 1 μL/min. Simultaneously, the high-precision vacuum pump, operating at a flow rate of 1.2 mL/min, was connected to the two GCGGM outlets, drawing air and formaldehyde gas into the gas channels of the chip and spontaneously generating an increasing formaldehyde concentration gradient from Line 1 to Line 4. Line 1 is assumed to contain only air and to be essentially free of formaldehyde gas.

Upon completion of the formaldehyde exposure experiment, the chip underwent a continuous 24 h dynamic cultivation period. Cell viability within each channel was subsequently assessed using the Live/Dead assay kit. As illustrated in Figures 3B,C, three locations within each channel were randomly selected for observation. The results indicated uniform cell viability across each channel, with a gradual decline in viability observed in response to increasing formaldehyde concentrations.

### Gene expression profiles following exposure to different concentrations of formaldehyde

**FIGURE 4:**
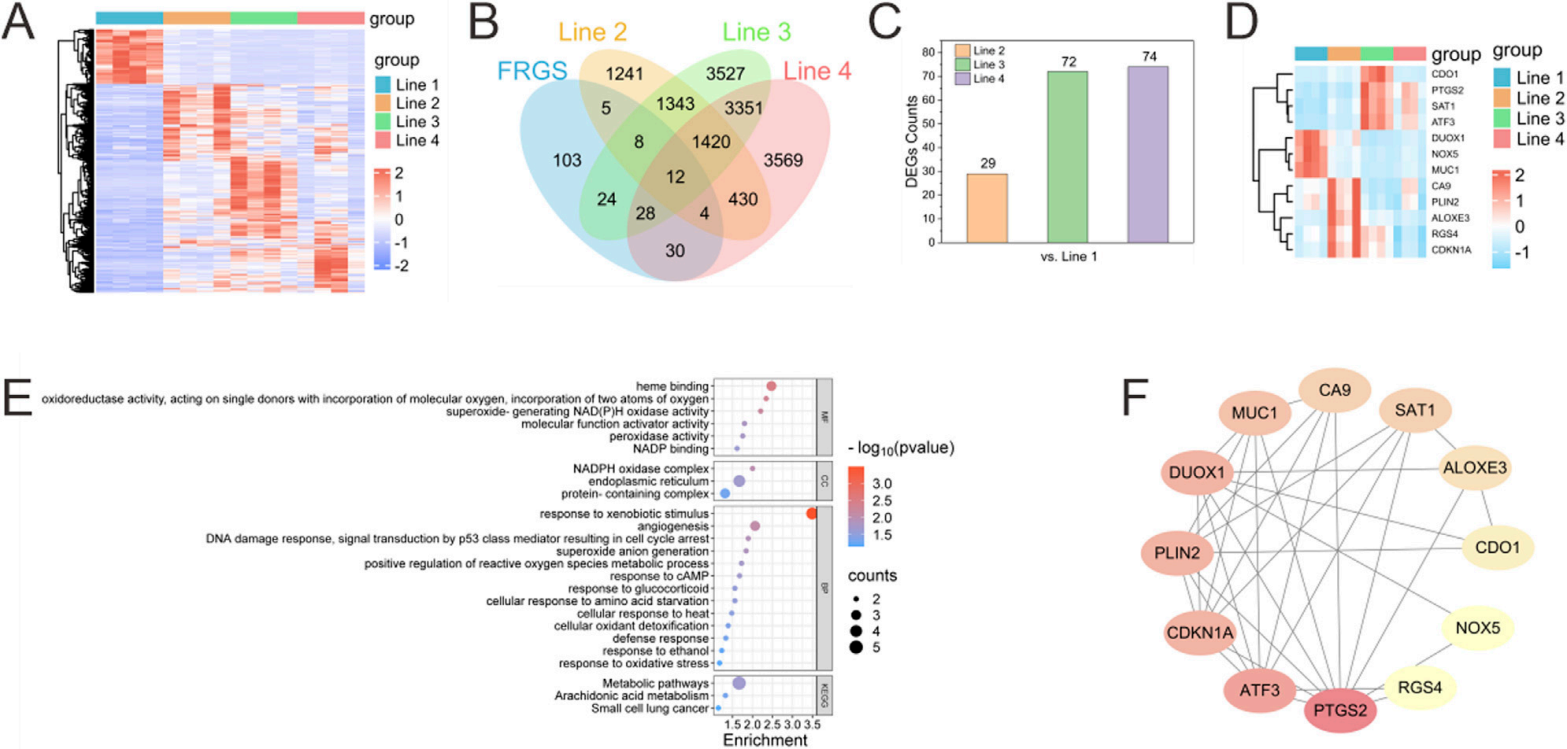
Bioinformatics analysis of changes in the transcriptome of BEAS-2B cells after exposure to formaldehyde. **(A)** Heatmap of differentially expressed genes (DEGs) across different Lines compared to Line 1 (red representing upregulated and blue representing downregulated); **(B)** Venn diagram illustrates the number of intersecting DEGs from disparate lines that intersect with the FRGS; **(C)** Number of DEGs in different Lines overlapping with FRGR; **(D)** Heatmap of the 12 overlapping FRGS across different Lines **(E)** GO functional classification and KEGG pathway annotation for the 12 overlapping FRGs in the Venn diagram **(F)** PPI network of the 12 overlapping FRGS.

Figure 4A presents a heatmap demonstrating the expression characteristics of all differentially expressed genes (DEGs) across the four groups (Line 1 to Line 4). Compared to the Line 1 group (which was free of formaldehyde), it was observed that there were significant differences in the expression patterns and trends of DEGs in the Line 2 to Line 4 groups (which were exposed to formaldehyde). The FerrDb database (http://www.zhounan.org/ferrdb/) was searched to identify 214 ferroptosis-related genes (FRGS). Intersecting the DEGs from the formaldehyde exposure groups with the FRGS yielded 29 overlapping genes in the Line 2 group, 72 in the Line 3 group, and 74 in the Line 4 group. The increasing number of overlapping DEGs with higher concentrations of formaldehyde exposure suggests a progressive activation of FRGS as formaldehyde concentration increases (Figures 4B,C). Additionally, 12 overlapping FRGS were commonly expressed across all three formaldehyde-exposed groups (Figure 4D).

### Enrichment analysis and protein-protein interaction construction of overlapping FRGS

Enrichment analysis of KEGG and GO annotation were conducted to elucidate the biological classification and function of the 12 overlapping FRGS (Figure 4E). GO annotation categorized these genes into three main domains: biological process (BP), cellular component (CC), and molecular function (MF). The molecular functions (MF) associated with ferroptosis included “oxidoreductase activity, acting on single donors with incorporation of molecular oxygen, incorporation of two atoms of oxygen,” “superoxide-generating NAD(P)H oxidase activity,” and “peroxidase activity.” The biological processes (BP) linked to ferroptosis involved “positive regulation of reactive oxygen species metabolic process,” “response to oxidative stress,” and “cellular oxidant detoxification.” KEGG analysis of the overlapping FRGS revealed alterations in multiple pathways induced by formaldehyde exposure, including “Metabolic pathways,” “Arachidonic acid metabolism,” and “Small cell lung cancer.”

A protein–protein interaction (PPI) network was constructed for the 12 overlapping FRGS using the online STRING database, revealing interactions among these genes (Figure 4F). Hub genes were identified by means of Cytoscape, which was employed to calculate the node degree using the CytoHubba plugin. This analysis identified the top 5 hub genes within the overlapping FRGS as *PTGS2*, *ATF3*, *CDKN1A*, *PLIN2*, and *DUOX1*.

### Effect of deferoxamine mesylate intervention on ferroptosis-related biomarkers triggered by formaldehyde-exposed on-chip

**FIGURE 5:**
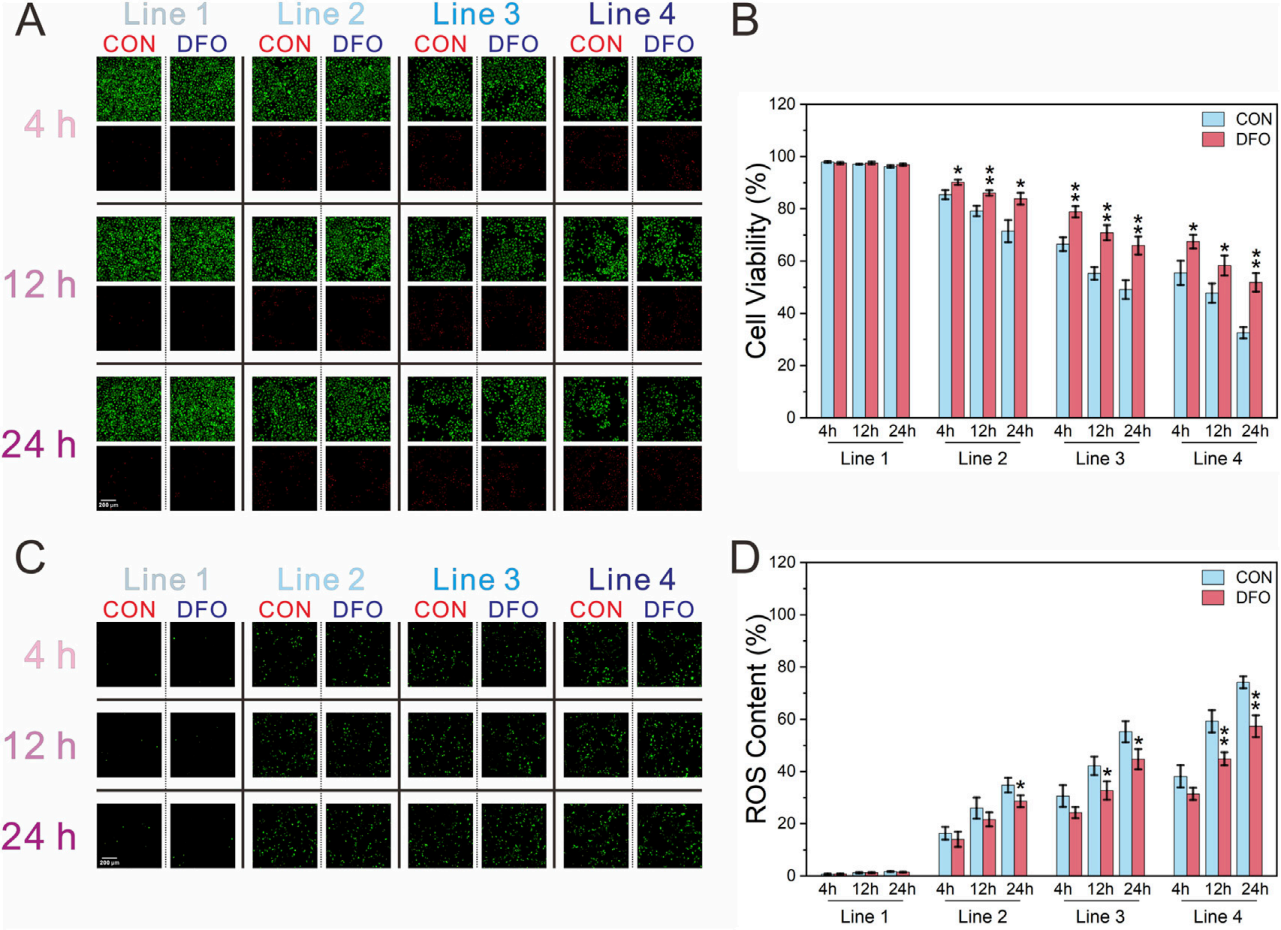
Assessment of cell viability and ROS content of BEAS-2B cells following exposure to formaldehyde. **(A,B)** Fluorescence images of cell viability and its quantification (green: live cells; red: dead cells); **(C,D)** Fluorescence images of ROS content and quantification of ROS normalized to cell viability; *P < 0.05, **P < 0.01 (n = 3).

Formaldehyde exposure in the chip impacted cell viability in a concentration- and time-dependent manner, with viability decreasing as formaldehyde concentration increased and the duration of post-exposure dynamic culture was extended. At most time points, the ferroptosis inhibitor deferoxamine mesylate (DFO) significantly mitigated cell death across all formaldehyde concentrations and post-exposure culture intervals (Figures 5A,B). Reactive oxygen species, key regulators of ferroptosis, were also evaluated (Figures 5C,D). Since the reactive oxygen species (ROS) fluorescent probe does not label dead cells, ROS levels were normalized to cell viability. In all formaldehyde exposure groups, ROS levels correlated positively with both formaldehyde concentration and the duration of dynamic culture following exposure. In Line 2, It was observed that in Line 2, DFO-exposed groups exhibited significantly lower ROS levels in comparison to the CON group only at 24 h post-exposure, whereas in Lines 3 and 4, the DFO group exhibited significantly reduced ROS levels compared to the CON group at both 12 and 24 h post-exposure.

**FIGURE 6:**
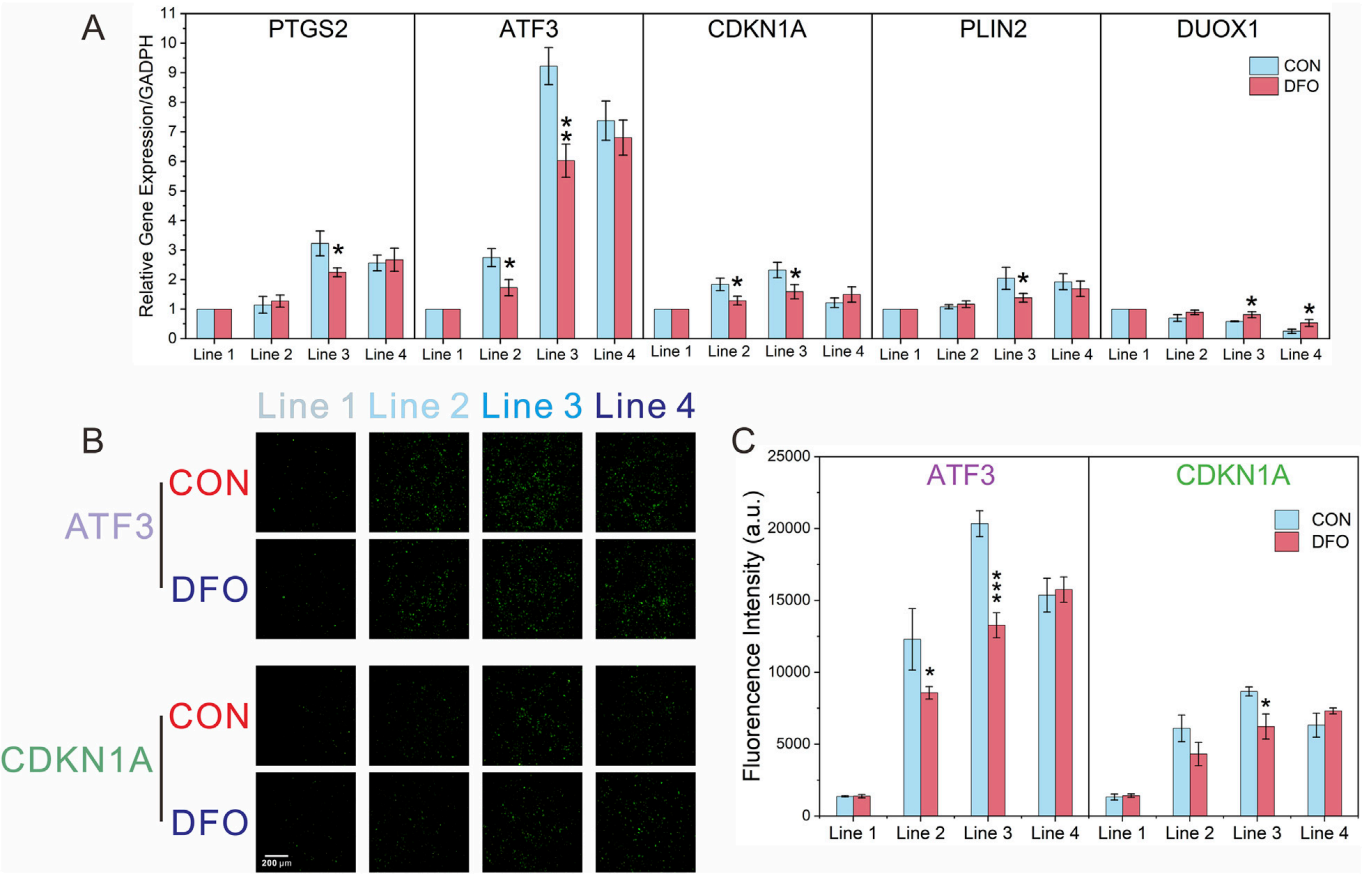
RT-qPCR and immunofluorescence analysis confirming the transcriptomic results. **(A)** Relative mRNA expression levels of ATF3 and CDKN1A after formaldehyde exposure, with and without DFO intervention, analyzed by RT-qPCR; **(B,C)** Immunofluorescence analysis of ATF3 and CDKN1A after formaldehyde exposure, with and without DFO intervention; Significance levels: *P < 0.05, **P < 0.01, ***P < 0.001 (n = 3).

Following the identification of the top five hub genes in the PPI network (*PTGS2*, *ATF3*, *CDKN1A*, *PLIN2*, and *DUOX1*), the validation process was initiated. This was achieved by RT-qPCR in order to confirm the effect of DFO on ferroptosis induced by formaldehyde exposure. As shown in Figure 6A, in the CON group, the mRNA levels of *PTGS2*, *ATF3*, *CDKN1A*, and *PLIN2* initially increased, followed by a subsequent decrease as formaldehyde exposure concentrations intensified. Furthermore, the mRNA level of *DUOX1* showed a consistent decline in response to increasing formaldehyde concentrations, which aligns with the results of the transcriptomic analysis. Additionally, compared to the CON group, the effects of DFO intervention demonstrated variability across different hub genes and formaldehyde concentrations. Specifically, in Line 2, DFO significantly impacted *ATF3* and *CDKN1A*. And in Line 3, it exhibited significant effects on all five hub genes. However, in Line 4, DFO significantly influenced only *DUOX1*.

With a view to investigating protein expressions at each formaldehyde concentration, experiments were conducted for on-chip detection of *ATF3* and *CDKN1A* using immunofluorescence. As shown in Figures 6B,C, the protein levels of *ATF3* and CDKN1A initially increased and then declined with increasing formaldehyde concentrations in the CON group. This trend aligns with the findings from transcriptomic analysis and RT-qPCR. Additionally, compared to the CON group, DFO significantly affected *ATF3* expression in Lines 2 and 3, and *CDKN1A* expression in Line 3.

## Discussion

Conventional cell culture techniques employed in the assessment of the toxic effects of gaseous pollutants are inherently limited in several ways (Tang et al., 2023). These techniques frequently rely on liquid media, yet the properties of certain gaseous pollutants result in their limited solubility in liquids (Tang et al., 2023). This may lead to insufficient exposure to the pollutants and thus compromise the accuracy of experimental outcomes. Furthermore, from a biomimetic perspective, conventional cell culture methods fail to replicate the physiological gas-liquid interface of the respiratory system, nor can they support dynamic cell culture to emulate the stable physiological conditions of the human body. To address the aforementioned challenges, recent years have witnessed the development of biomimetic lung chips based on microfluidic technology, offering a novel approach for the *in vitro* exposure of gaseous pollutants (Monteduro et al., 2023; Shrestha et al., 2020). These chips not only enable the study of cellular responses in a more physiologically relevant context but also facilitate the precise control of fluid flow to provide specific stimuli to cells (Ding et al., 2021). The emergence of modular microfluidic chips marks a major leap forward in the design and functional integration of microfluidic devices (Hsieh et al., 2014; Moukachar et al., 2023; Nie et al., 2018). In this study, the completed assembled modular system enables the execution of conduct gas exposure experiments, and facilitates the independent collection of cellular samples at different formaldehyde concentrations through the separation of the GCGGM from the CCM (Figure 1).

Understanding formaldehyde toxicity is critically important in both clinical (Lam et al., 2021; Xu et al., 2024) and environmental contexts (Cammalleri et al., 2022). Formaldehyde is a widespread environmental pollutant with adverse effects for human health, particularly concerning respiratory diseases and carcinogenesis. Our modular biomimetic lung chip enables a more accurate and reproducible assessment of formaldehyde-induced toxicity, providing insights that are more relevant to real-world exposures. By utilizing this platform, researchers can study formaldehyde’s effects under dynamic, controlled conditions that closely replicate human lung physiology, making it a powerful tool for assessing environmental pollutants and advancing toxicological research.

To verify the feasibility and convenience of stepwise assembly of the chip for *in situ* gas exposure and cell sample collection, BEAS-2B cells within the chip were exposed to a formaldehyde concentration gradient. Cell samples from each gas channel were collected via *in situ* lysis and subsequently subjected to transcriptomic sequencing. Gene expression analysis revealed alterations in the transcriptome of BEAS-2B cells exposed to varying concentrations of formaldehyde (Figure 4A). The increasing number of DEGs overlapping with FRGS as formaldehyde concentration increased suggests a progressive activation of ferroptosis-related pathways in the cells (Figures 4B,C). Notably, 12 overlapping FRGS were consistently expressed across all exposure levels, indicating their potential as biomarkers for formaldehyde-induced ferroptosis (Figure 4D). GO and KEGG pathway enrichment analyses highlighted key processes such as oxidative stress response (Figure 4E), ROS metabolism, and cellular oxidant detoxification, which are central to ferroptosis regulation (Xiang et al., 2024). The PPI network analysis further identified key hub genes, including *PTGS2*, *ATF3*, *CDKN1A*, *PLIN2*, and *DUOX1*, which are potentially linked to formaldehyde-induced ferroptosis (Figure 4F) (Chen et al., 2024; Kang et al., 2019; Wang et al., 2021). These hub genes play significant roles in crucial pathways such as oxidative stress response, inflammation, and cell cycle regulation.

To determine whether exposure to different concentrations of formaldehyde at the gas-liquid interface induces ferroptosis, the ferroptosis inhibitor DFO was introduced to assess its effects on cell viability and the ferroptosis biomarker, ROS (Li et al., 2021). The results revealed a decline in cell viability with increasing formaldehyde concentrations and prolonged exposure during continuous dynamic culture. At various time points during continuous dynamic culture post-exposure, DFO significantly reversed the decrease in cell viability caused by formaldehyde exposure at different concentrations (Figures 5A,B). Furthermore, an increase in intracellular ROS levels was observed, which was more pronounced at higher formaldehyde concentrations and during longer exposure periods (Figures 5C,D). Among all formaldehyde concentrations, during the first 4 h of continuous dynamic culture post-exposure, DFO did not significantly inhibit the rise in ROS compared to the CON group. However, after 12 h of continuous dynamic culture post-exposure, DFO significantly reduced ROS levels in both Line 3 and Line 4, and at 24 h of dynamic culture, it was able to suppress ROS elevation across all formaldehyde concentrations. The findings suggest that formaldehyde exposure can induce ferroptosis in BEAS-2B cells at the gas-liquid interface through both concentration- and time-dependent effects.

The validation of the DEGs through RT-qPCR and immunofluorescence confirmed the reliability of the transcriptomic analysis and further established the role of ferroptosis-related biomarkers in formaldehyde-induced toxicity (Figure 6). The observed trends in mRNA and protein levels, including the *ATF3* and *CDKN1A*, followed an increasing and then decreasing pattern at progressively higher concentrations of formaldehyde. *ATF3* plays a critical role in the cellular response to stress and injury (Wang et al., 2021). As a transcription factor, it regulates signal transduction and coordinates multiple pathways, including apoptosis, ferroptosis, and cellular differentiation. Additionally, *ATF3* acts as a mediator between inflammation, oxidative stress, and immune responses (Wang et al., 2021). The *CDKN1A* gene, or p21, is an important cyclin-dependent kinase inhibitor (CDKI) that regulates cell cycle progression by inhibiting cyclin-dependent kinases (CDKs), thereby halting cell cycle progression. During ferroptosis, oxidative stress activates intracellular stress response pathways, leading to changes in *CDKN1A* expression (Kang et al., 2019). By modulating cell cycle arrest, CDKN1A influences the cell’s metabolic state and indirectly impacts processes linked to ferroptosis, such as intracellular iron metabolism and antioxidant systems. In summary, we hypothesize that formaldehyde-induced ferroptosis may occur through CDKN1A, which inhibits CDK2 activity and arrests the cell cycle at the G1 phase. This leads to enhanced autophagic degradation of ferritin, releasing excessive free iron (Fe^2+^), which, through the Fenton reaction, catalyzes the generation of hydroxyl radicals from H_2_O_2_, thereby increasing ROS levels. Meanwhile, ATF3, as a stress response factor, suppresses the Nrf2/ARE signaling pathway (downregulating the expression of glutamate-cysteine ligase subunits GCLC/GCLM), reduces glutathione (GSH) synthesis, and inhibits SLC7A11 expression, thus blocking cysteine uptake. This results in GSH depletion, inactivation of GPX4, and a loss of the cell’s ability to repair lipid peroxidation.

The validation of the DEGs through RT-qPCR and immunofluorescence confirmed the reliability of the transcriptomic analysis and further reinforced the role of ferroptosis-related biomarkers in formaldehyde-induced toxicity. These findings suggest that formaldehyde exposure may initially activate a stress response aimed at repairing cellular damage, but sustained exposure may ultimately lead to ferroptosis.

Due to the modular chip design and stepwise assembly approach, the results of this study provide valuable insights into formaldehyde-induced ferroptosis. While formaldehyde has long been linked to respiratory irritation and toxicity, the involvement of ferroptosis in these conditions has remained underexplored (Bernardini et al., 2022). This study demonstrates the activation of ferroptosis-related pathways in lung cells exposed to formaldehyde, revealing a potential new mechanism of formaldehyde toxicity that may contribute to respiratory diseases through ferroptosis. Furthermore, the use of the modular biomimetic lung chip offers a new tool for studying the effects of airborne toxins, presenting a promising alternative to traditional experiments.

While this study provides valuable insights into formaldehyde-induced ferroptosis and the new modular biomimetic lung chip, several areas require further investigation. First, expanding the study to encompass a broader range of formaldehyde concentrations and exposure durations would provide a more comprehensive understanding of the dose-response relationship and the long-term effects of formaldehyde on lung cells. Additionally, investigating the role of other ferroptosis-related genes and proteins could uncover potential therapeutic targets to mitigate formaldehyde-induced toxicity. Lastly, this study did not utilize primary cells to better simulate the lung gas-blood barrier. Future research may incorporate more complex cell models, such as co-culture of various lung cell types or the use of primary cells to construct the model, in order to more accurately replicate the *in vivo* microenvironment and investigate the intercellular interactions that contribute to formaldehyde-induced toxicity.

## Materials and methods

### Design

The modular chip comprises two modules (Figure 1): the CCM and the GCGGM. The CCM is composed of three distinct layers, the upper layer comprising four gas channels measuring 2 mm in width and 0.8 mm in depth, which are designated for cell loading and gas exposure, and the lower layer consisting of four liquid channels measuring 1 mm in width and 0.15 mm in depth. The CCM also contains a PC membrane, with a pore diameter of 5 µm (TMTP09030, Isopore, Ireland), is sandwiched between the upper and lower layers; the alignment of these layers is facilitated by eight alignment holes per layer, ensuring precise assembly during the bonding process. The GCGGM consists of 2 identically structured single-layer; each module features a horizontal channel measuring 2 mm in width and 0.8 mm in depth, accompanied by 4 alignment micropillars. During the assembly process with the CCM, the horizontal channels of the GCGGM are seamlessly connected to the 4 inlets and 4 outlets of the gas channels in the upper layer through 4 micropillars and pressure-sensitive adhesive, achieving fast and precise self-aligned integration.

### Fabrication of the chip

The fabrication of both the upper and lower layers of the CCM and the GCGGM was accomplished through the utilization of PDMS (Sylgard 184, DOW, Wiesbaden, Germany). The lower layer of the CCM was processed via soft lithography, while the upper layer and the GCGGM were manufactured by casting the aluminum mold (MesoBioSys Co., Ltd, Hubei, China) with PDMS. A PC membrane was cut into predefined shapes using a CO_2_ laser (VSL2.30, Universal, PA, United States), then rinsed 3 times with deionized water and dried with nitrogen gas. The PC membranes were activated in an oxygen-filled plasma chamber (500 mTorr, 100 W, DT-01, Suzhou OPS Plasma Technology Co., Jiangsu, China) for 1 min and immediately immersed in a 5% 3-aminopropyltriethoxysilane (APTES, A3648, Sigma-Aldrich, MO, United States) aqueous solution at 80 °C. After 20 min, the membranes were removed from the APTES solution, rinsed 3 times with deionized water, and the fabricated lower PDMS layer was continuously activated in an oxygen-filled plasma chamber for 30 s (500 mTorr, 100 W). Finally, the membranes were gently contacted and aligned with the lower layer of the CCM and immediately placed at 80 °C for 2 h. After completing the aforementioned steps, the composite structure and the upper layer of CCM were activated in the same oxygen-filled plasma chamber, gently contacted and pressed together, and subsequently placed at 80 °C. A 200 g metal block was carefully pressed onto the sandwich structure for 2 h.

Prior to assembling the GCGGM and CCM, the pressure-sensitive adhesive was precisely cut using a CO_2_ laser, ensuring a perfect match to the GCGGM’s shape and incorporating 4 alignment holes. Within an ultra-clean bench, the adhesive was affixed to the underside of the GCGGM and exposed to ultraviolet light for 2 h to sterilize its surface. Subsequently, the adhesive was integrated onto the upper surface of the CCM’s upper layer via the GCGGM’s alignment micropillars. Thus, the complete assembly of the chip was successfully finalized.

### Functional characterization of the chip

The exposure system was constructed by applying negative pressure to outlets A and B of the GCGGM, thereby drawing gases A and B from inlets A and B into the gas channels of the upper layer of the CCM (flow rate: 1.2 mL/min). This setup created a gas concentration gradient through free diffusion. The injection of liquid into the four liquid channels of the CCM was achieved using a syringe pump (TS-1B, LongerPump, China) at a rate of 1 μL/min. For validation, gas A was designated as air and gas B as CO_2_, and the CO_2_ concentration gradient was evaluated using a bromothymol blue pH indicator solution (pH range: 6–8). After stabilising the liquid flow, air and CO_2_ were directed to inlets A and B of the GCGGM, respectively, while maintaining steady negative pressure (OB1 MK3, Elveflow, French) at outlets A and B to regulate the gas flow. The principle of using the CO_2_ and bromothymol blue colorimetric reaction to characterize the gas concentration gradient is that CO_2_, when dissolved in water, forms carbonic acid, thereby lowering the pH. This acidic environment induces a color change in the pH indicator bromothymol blue, shifting from blue to yellow. The lower the CO_2_ concentration, the more bluish the bromothymol blue becomes, whereas higher concentrations result in a more yellowish color. The alterations in the color of the pH indicator were recorded and subjected to thorough analysis using ImageJ.

To validate the stability of the gas-liquid interface within the chip and determine whether liquids in the lower layer channels might overflow and induce liquid crosstalk, a syringe pump was used to infuse different colored inks into each liquid channel of the lower chip at a flow rate of 1 μL/min. Observations were conducted and recorded to ascertain whether the colors from distinct channels intermixed.

### Cell loading and culturing

The BEAS-2B human bronchial epithelial cell line was obtained from the American Type Culture Collection (United States). The cells were cultured in RPMI 1640 medium (11875-093, Gibco, United States) supplemented with 10% fetal bovine serum (10100147, Gibco, United States), 100 IU/mL penicillin, and 100 μg/mL streptomycin (15140-122, Gibco, United States). For further information regarding culture methods for BEAS-2B cells, please refer to the previously published articles (Li et al., 2024; Xue et al., 2023). The sterilization of the modular chip is achieved through the following method: the CCMs were exposed to UV light for a period of two hours within an ultra-clean bench, and the subsequent step is a series of rinses, commencing with 75% alcohol and concluding with sterile water. In order to enhance cell adhesion, collagen I (150 μg/mL) (354249, Corning, United States) was incorporated into the upper and lower channels of the CCMs and subsequently incubated in a cell incubator for a duration of 12 h. The BEAS-2B cells were prepared at a concentration of 3 × 10^6^ cells/mL and subsequently infused through the gas channel inlets at a flow rate of 50 μL/min using a syringe pump. Following this, the cells were seeded onto the microporous membrane of the CCM and then incubated in the cell incubator (HERAcell 240, Thermo Scientific, Germany).

### Experimental procedure of chip operation and formaldehyde exposure

The medium was carefully aspirated from the gas channel 2 h prior to the commencement of the formaldehyde exposure experiment. Thereafter, the medium (CON group) or the medium containing the ferroptosis inhibitor deferoxamine mesylate (50 μM, DFO group; DFO, HY-B0988, United States) was introduced into the lower-layer channels at a flow rate of 1 μL/min using a syringe pump. A 0.4 mL of 38% formaldehyde solution was added to a 4 L gas sampling bag containing 2 L of sterile air and left undisturbed at 25 °C for 2 h. Prior to the exposure experiment, the GCGGM was securely affixed to the CCM using pressure-sensitive adhesive and self-aligning micropillars to ensure a tight, leak-proof seal. A high-precision vacuum pump was connected to outlets A and B of the GCGGM via conduits, while the air bag (containing 2 L of sterile air) and the formaldehyde gas bag were quickly connected to inlets A and B, respectively. The exposure experiment was conducted in an ultra-clean bench and lasted for 12 min.

Upon completion of the exposure experiment, the GCGGM was carefully detached from the surface of the CCM. Thereafter, a transfer of the chip was conducted to a cell culture incubator, where it underwent continuous dynamic cultivation over a 24-h period.

### Transcriptome analysis, and RT-qPCR

To access further detailed methods for RNA extraction for BEAS-2B cells, please refer to the relevant publications (Li et al., 2024). Following the subjection of total RNA to mRNA purification employing Oligo (dT)-attached magnetic beads, the resultant mRNA was fragmented into smaller pieces. The fragments were then reverse transcribed and validated for quality control purposes, thus yielding the final sequencing library. The purification of PCR products was accomplished through the utilisation of the AMPure XP system (Beckman Coulter, United States), while the assessment of library quality was conducted on an Agilent Bioanalyzer 4150 system (Agilent, United States).

In the present study, the Roche Transcriptor First Strand cDNA Synthesis Kit (04896866001, Roche, Germany) was utilised for the reverse transcription of purified RNA. The subsequent RT-qPCR was performed in accordance with the manufacturer’s instructions using the FastStart Essential DNA Green Master Kit (06924204001, Roche, Germany) in the LightCycler^®^ 96 Instrument (Roche, Germany). As outlined in Table 1, the primer sequences for each gene, synthesised by Sangon Biotech (Shanghai, China), are presented in detail. Conditions were as follows: Initial denaturation at 95°C for 15 min, followed by 40 cycles of 30 s at 95°C, 1 min at 55°C, and 30 sat 72°C. The expression levels were then normalized to Glyceraldehyde-3-phosphate dehydrogenase (*GAPDH*) using Equation 2^−ΔΔCt^.

**TABLE 1:** Primer sequences for RT-qPCR.

Gene	Sequence
PTGS2	F: TGTACGGGGTTTGTGACTGG R: ACGAAGCATCCACAGATCCC
ATF3	F: CCTCTGCGCTGGAATCAGTC R: TTCTTTCTCGTCGCCTCTTTTT
CDKN1A	F: TGTCCGTCAGAACCCATGC R: AAAGTCGAAGTTCCATCGCTC
PLIN2	F: ATGGCATCCGTTGCAGTTGAT R: GGACATGAGGTCATACGTGGAG
DUOX1	F: AAGTTCGACCTCAGGACCACTAT R: GGGAGTTGAAGAAGGGCTCAAAG
GAPDH	F: GGAGCGAGATCCCTCCAAAAT R: GGCTGTTGTCATACTTCTCATGG

### Bioinformatics analysis

Bioinformatics analysis was performed using the online platform Dr. Tom (BGI Company, Beijing, China). Four biological replicates were included for each concentration group in the experiment. Raw reads in FASTQ format generated by Illumina were trimmed to remove low-quality ends (phred score <30) and short read lengths (minimum length = 30), yielding clean reads. Clean reads were aligned to the hg38 reference genome using STAR (https://github.com/alexdobin/STAR). Gene expression levels were estimated using the FPKM method, and Pearson correlation coefficients were calculated to evaluate inter-sample correlations in gene expression levels (*R* ^2^ > 0.8). Following gene expression quantification via featureCounts tool, data normalization and differential expression analysis were performed using DESeq2. A negative binomial distribution model was applied, and the Benjamini–Hochberg method was employed for multiple hypothesis testing to control the false discovery rate (FDR). Differentially expressed genes were identified using an adjusted p-value threshold (*P*.adj <0.05) and a minimum fold-change cutoff of 2-fold (|log_2_ (fold change)| > 1.0).

Gene Ontology (GO) and Kyoto Encyclopaedia of Genes and Genomes (KEGG) were utilised to perform analyses in order to gain insights into phenotypic changes, with the R Phyper function being employed for this purpose. Furthermore, exploratory analyses were conducted on the primary functions of DEGs. A PPI network was constructed using STRING (https://string-db.org/), with the hub genes identified based on their degrees using CytoScape 3.7.1.

### Assessment of cell viability and reactive oxygen species using fluorescence imaging

Assessment of cell viability was conducted utilizing a Live/Dead assay kit (L3224, Invitrogen, United States) (Li et al., 2024). The production of ROS was measured using a ROS assay kit (R252, Dojindo, Japan) (Li et al., 2024). Preparation of all working solutions was conducted in strict accordance with the manufacturer’s guidelines. Subsequent to formaldehyde exposure, the chips were rinsed thrice with Hank’s Balanced Salt Solution (HBSS) for a duration of 3 minutes on each occasion. Then, the working solution was injected into the chip using a syringe pump and the chip was left to incubate for 20 min. Finally, the chip was subjected to a further rinse with HBSS for 15 min and then subjected to imaging using an inverted fluorescence microscope (IX71, Olympus, Japan).

### Immunocytochemistry

The immunocytochemistry procedure was conducted in accordance with the following protocol: The BEAS-2B cells, which had been cultured on-chip, were fixed with 4% paraformaldehyde in PBS for 15 min at room temperature (RT). Following this, permeabilization and blocking was carried out on the cells using PBS containing 0.3% Triton X-100 (A110694, Sangon Biotech, China) and 5% bovine serum albumin for a period of 20 min at RT. Following this, an overnight incubation was conducted with rabbit anti-human *Z O -1* (ab221547, Abcam, United States), anti-human *ATF3* (ab207434, Abcam, United States), and anti-human *p21* (ab109520, Abcam, United States) primary antibodies, dissolved in PBS containing 5% bovine serum albumin. To facilitate visualization, a secondary antibody conjugated with goat anti-rabbit IgG H&L Alexa Fluor 488 (ab150077, Abcam, United States) was utilized. Nuclei were counterstained with DAPI (D9542, Sigma-Aldrich, Israel) at a concentration of 0.5 μg/mL in PBS. The subsequent capture of fluorescent images was facilitated by means of a confocal laser scanning microscope (FV3000, Olympus, Japan).

### Image and data analysis

A confocal laser scanning microscope (FV3000, Olympus, Japan) and an inverted fluorescence microscope (IX71, Olympus, Japan) were utilised for image capture. Subsequent image analysis was performed using ImageJ, and statistical analysis was conducted with SPSS 17.0 (SPSS Inc., United States). The experimental results were derived from at least three independent replicates and expressed as mean ± standard deviation (SD). Statistical analyses were performed using the independent samples t-test and one-way analysis of variance (ANOVA), with all datasets verifying assumptions through Levene’s test (homogeneity of variance) and the Shapiro-Wilk test (normality). For significant differences identified by ANOVA (*P* < 0.05), *post hoc* multiple comparisons were conducted using the LSD and Tamhane’s T2 methods.

## Conclusion

This study aims to develop a stepwise-assembled modular biomimetic lung chip and utilize this chip, along with the stepwise assembly experimental approach, to investigate formaldehyde-induced ferroptosis. The goal is to demonstrate the chip’s applicability in gas pollutant exposure, biomarker detection, and analytical experiments. The results showed that the chip’s cell culture module and gas concentration gradient generation module could rapidly align through micro-pillars and alignment holes, forming a stable gas-liquid interface and gas concentration gradient. Moreover, through the stepwise assembly approach, the chip can easily facilitate experiments related to cell loading, gas concentration gradient exposure, and cell sample collection. The application of this chip has revealed the critical role of formaldehyde in activating ferroptosis-related pathways.

## Data Availability

The data presented in the study are deposited in the NCBI repository, accession number SRR32342750 ∼ SRR32342765 (BioProject: PRJNA1223810).
